# Open‐Window Mapping for Accessory Pathway Ablation: Influence of Catheter Design on Mapping Performance

**DOI:** 10.1002/joa3.70402

**Published:** 2026-06-19

**Authors:** Kazuma Iimura, Atsuhiko Yagishita, Aika Iijima, Mari Amino, Yuji Ikari, Koichiro Yoshioka

**Affiliations:** ^1^ Department of Cardiology Tokai University School of Medicine Isehara Japan

**Keywords:** accessory pathways, catheter ablation, open‐window mapping, Wolff–Parkinson–White syndrome

## Abstract

**Background:**

Open‐window mapping (OWM) using the extended early‐meets‐late (EEML) algorithm is widely used for accessory pathway (AP) localization. However, the impact of catheter design on mapping performance and procedural outcomes remains unclear.

**Methods:**

We retrospectively analyzed 43 consecutive patients undergoing OWM‐guided AP ablation. Twenty‐four patients were mapped using an 8‐spline high‐density catheter and 19 using a conventional 5‐spline catheter. Mapping characteristics and ablation outcomes were compared.

**Results:**

The 8‐spline catheter was associated with higher mapping density (7974 [5618–11 017] vs. 2485 [734–12 577] points; *p* < 0.001) and shorter mapping time (11.0 [8.5–14.8] vs. 28.0 [15.0–40.0] minutes; *p* < 0.001). The EEML‐derived AP gap width was narrower in the 8‐spline group (6.1 [5.3–7.5] vs. 8.3 [2.9–15.6] mm; *p* = 0.023), with lower EEML thresholds required for visualization (*p* = 0.003). First‐pass AP elimination was achieved in all patients in the 8‐spline group (100%) compared with 68% in the 5‐spline group (*p* = 0.004), with fewer radiofrequency applications required (*p* = 0.048). Fluoroscopy time and complication rates were similar between groups.

**Conclusion:**

Compared with a conventional 5‐spline catheter, an 8‐spline high‐density catheter was associated with improved mapping efficiency and a higher rate of first‐pass ablation success. These findings suggest that catheter design may influence procedural performance in OWM‐guided AP ablation.

## Graphical abstract

8‐spline high‐density open‐window mapping achieved higher spatial resolution, sharper EEML‐defined accessory pathway gap delineation, and shorter mapping time compared with 5‐spline catheters, and was associated with higher first‐pass ablation success, supporting improved mapping‐guided targeting precision in Wolff–Parkinson–White syndrome.

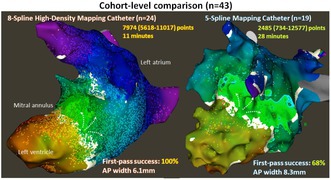

**Abbreviations**
3D: three‐dimensional
AP: accessory pathway
LA: left atrium/left atrial
OWM: open‐window mapping
RA: right atrium/right atrial
WPW: Wolff–Parkinson–White

## 1 Introduction

Accessory pathways (APs) bypass the normal atrioventricular conduction system and can lead to tachyarrhythmias requiring catheter ablation. Catheter ablation is an established first‐line therapy with high success rates and low procedural risk [1, 2, 3]. Successful ablation depends on precise localization of the AP insertion site. Conventional point‐by‐point mapping requires careful electrogram interpretation and may be operator‐dependent [4]. Three‐dimensional electroanatomical mapping systems have improved anatomical visualization and activation analysis, facilitating more efficient AP localization [5, 6].

Open‐window mapping (OWM) enables automated delineation of the conduction interface between early‐ and late‐activated tissue [7, 8, 9]. The extended early‐meets‐late (EEML) algorithm further enhances visualization by highlighting the transition between early and late activation, producing a characteristic gap corresponding to the AP conduction interface [10, 11, 12]. The spatial fidelity of this approach may depend on the sampling characteristics of the mapping catheter. Compared with conventional 5‐spline configurations, newer 8‐spline high‐density catheters incorporate additional splines, smaller electrodes, and closer interelectrode spacing, which may improve both near‐field signal resolution and spatial sampling density. Previous studies have shown that 8‐spline catheters acquire substantially more mapping points in shorter mapping times, suggesting improved mapping efficiency [13, 14, 15]. Although several case reports have suggested potential advantages of the 8‐spline catheter for OWM [16, 17, 18], whether these design differences translate into improved mapping fidelity and procedural outcomes during OWM‐guided AP ablation has not been systematically evaluated. Therefore, we investigated the association between catheter architecture and mapping performance during OWM‐guided AP ablation.

## 2 Materials and Methods

### 2.1 Study Population

This study included consecutive patients who underwent catheter ablation for APs using OWM between June 2021 and September 2025. The study was conducted in accordance with the Declaration of Helsinki and approved by the Institutional Ethics Committee of Tokai University Hospital (approval no. 25R135). Written informed consent was obtained from all patients.

A total of 43 patients who underwent AP ablation using the CARTO 3 system (Biosense Webster, Diamond Bar, CA, USA) were included. Patients were eligible if they had documented pre‐excitation on 12‐lead electrocardiography or electrophysiological confirmation of AP conduction and underwent OWM using either an 8‐spline or 5‐spline multipolar mapping catheter. Twenty‐four patients were mapped using an 8‐spline high‐density catheter (8‐spline group), and 19 using a 5‐spline catheter (5‐spline group). Catheter selection followed a sequential adoption pattern: the 5‐spline catheter was used during the earlier phase of the study period, and the 8‐spline catheter was adopted thereafter and used exclusively. Accordingly, this comparison represents a time‐based cohort analysis rather than randomized allocation.

### 2.2 Catheter Ablation Procedure

Electrophysiological studies were performed via right femoral venous access. Diagnostic catheters were positioned in the right atrium, His bundle region, coronary sinus, and right ventricle to confirm AP conduction and characterize pathway properties. For left free‐wall APs, trans‐septal access was obtained using an RF needle and long sheath, followed by heparin administration to maintain an activated clotting time of 300–350 s. Procedures were performed by multiple operators; however, a single experienced attending electrophysiologist provided consistent procedural oversight throughout the study period. Mapping settings and procedural workflow were unchanged during the study.

Radiofrequency ablation was performed using an irrigated contact force–sensing catheter (QDOT Micro, Biosense Webster) via a steerable sheath. Ablation targets were selected based on the EEML gap and earliest activation during retrograde or antegrade conduction. Microelectrode signals from the ablation catheter were not used for mapping interpretation or target selection. RF energy was delivered at 30–35 W with 30 mL/min irrigation and a temperature limit of 43°C, targeting an ablation index of 450. Successful ablation was defined as complete elimination of AP conduction, including disappearance of pre‐excitation (for manifest APs), non‐inducibility of atrioventricular reentrant tachycardia, and absence of retrograde conduction. The number of RF applications required was recorded, and first‐pass success was defined as complete elimination of AP conduction with the initial RF application.

### 2.3 Open‐Window Mapping and Catheter Design

Three‐dimensional electroanatomical mapping was performed using the CARTO 3 system (Version 7, Biosense Webster) with either the Pentaray catheter (5 splines, 4 electrodes per spline, 20 electrodes total, 2–6–2‐mm spacing) or the Octaray catheter (8 splines, 3 electrodes per spline, 24 electrodes total, 2–2–2‐mm spacing). Automatic annotation was performed using the ConfiDENSE module. The TrueRef setting, available only with the 8‐spline mapping catheter, was enabled in all cases in the 8‐spline group to minimize unipolar signal drift and improve annotation stability. This feature was not available with the 5‐spline catheter. The window of interest was set to include both atrial and ventricular components. Manual re‐annotation was performed only when clear misannotation was identified, such as inappropriate annotation of far‐field signals or inconsistent timing relative to adjacent points.

OWM was constructed using the EEML algorithm. The lower EEML threshold was adjusted on a per‐patient basis to align the EEML‐derived gap with the propagation map and optimize visualization of the conduction interface. Threshold adjustment followed a standardized approach: (1) the lower threshold was initially set at a mid‐range value (typically around 15%); (2) it was then incrementally adjusted until the EEML gap spatially corresponded to the propagation‐defined conduction interface along the atrioventricular annulus; and (3) the selected threshold was confirmed when the EEML gap remained stable across consecutive beats. The EEML‐derived AP gap width was defined as the maximal extent of the visible EEML gap along the atrioventricular annulus.

**FIGURE 1:**
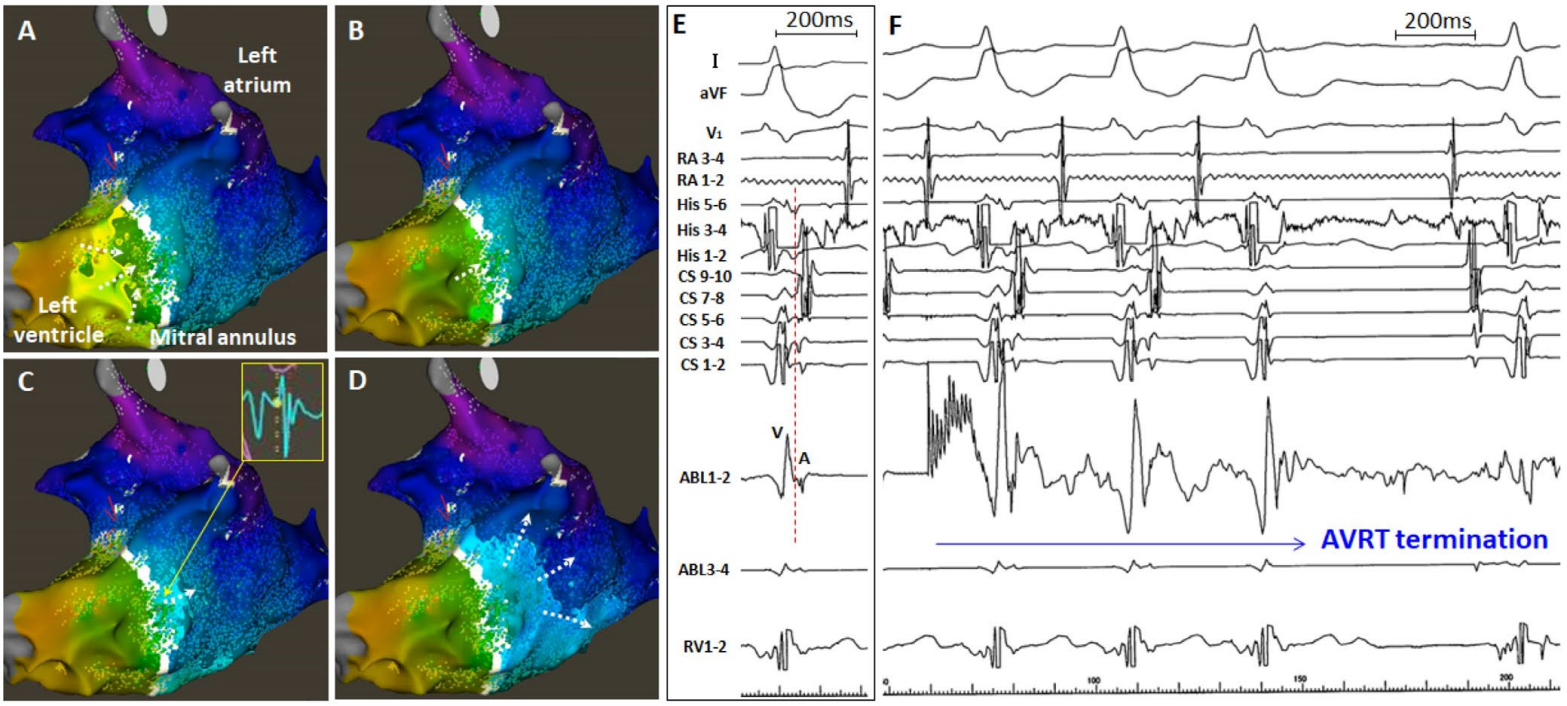
Representative case of open‐window mapping using an 8‐spline high‐density catheter in a patient with a concealed accessory pathway. (A–D) Open‐window mapping (OWM) performed during left ventricular pacing identified a posterolateral accessory pathway (AP) along the mitral annulus. The AP conduction gap was visualized by adjusting the lower extended early‐meets‐late (EEML) threshold to 19% to align with the propagation map (Supplementary Video 1). (E) Local electrograms recorded from the distal bipoles of the ablation catheter positioned within the EEML gap demonstrated the earliest atrial activation during ventricular pacing. (F) The first radiofrequency (RF) application at this site resulted in termination of atrioventricular reentrant tachycardia (AVRT) within 1 s. Small discontinuous white lines occasionally observed within the EEML gap represent either incomplete conduction block or threshold‐dependent segmentation artifacts and do not indicate separate APs. ABL, ablation catheter; AVRT, atrioventricular reentrant tachycardia; CS, coronary sinus; dist, distal; LV, left ventricle; prox, proximal; RA, right atrium; RF, radiofrequency.

**FIGURE 2:**
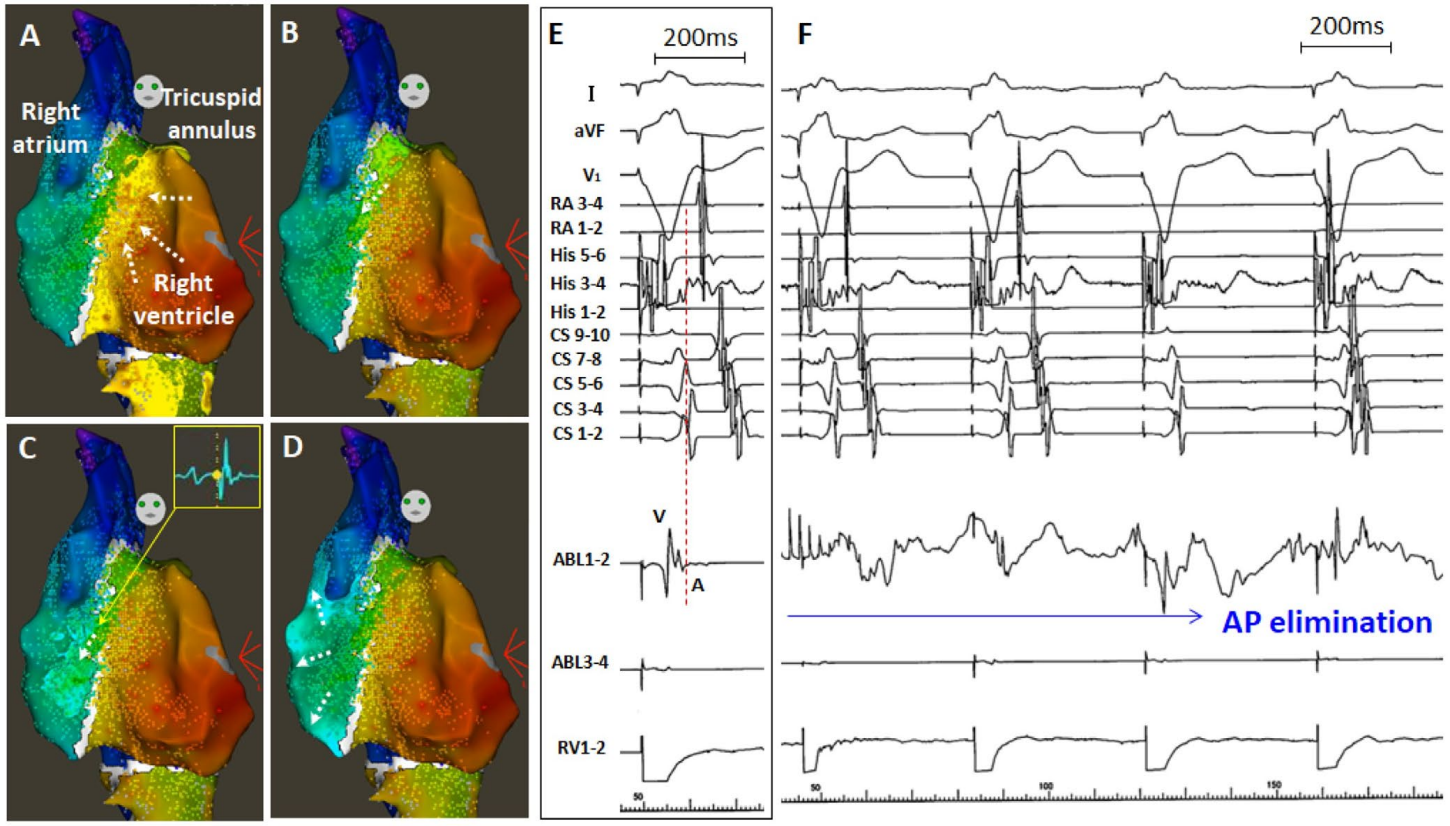
Representative case of open‐window mapping using an 8‐spline high‐density catheter (Octaray) in a patient with a concealed accessory pathway located at the lateral tricuspid annulus. (A–D) Open‐window mapping performed during right ventricular pacing identified a lateral accessory pathway (AP) along the tricuspid annulus. The AP conduction gap was visualized by adjusting the lower extended early‐meets‐late (EEML) threshold to 22% to align with the propagation map, which delineates oblique ventriculoatrial conduction across the tricuspid annulus (Supplementary Video 2). (E) Local electrograms recorded from the distal bipoles of the ablation catheter positioned within the EEML gap demonstrated the earliest atrial activation during ventricular pacing. (F) Following the first radiofrequency (RF) application, separation of previously fused atrial and ventricular components was observed at the distal bipoles, indicating successful elimination of AP conduction within 2 s.

**FIGURE 3:**
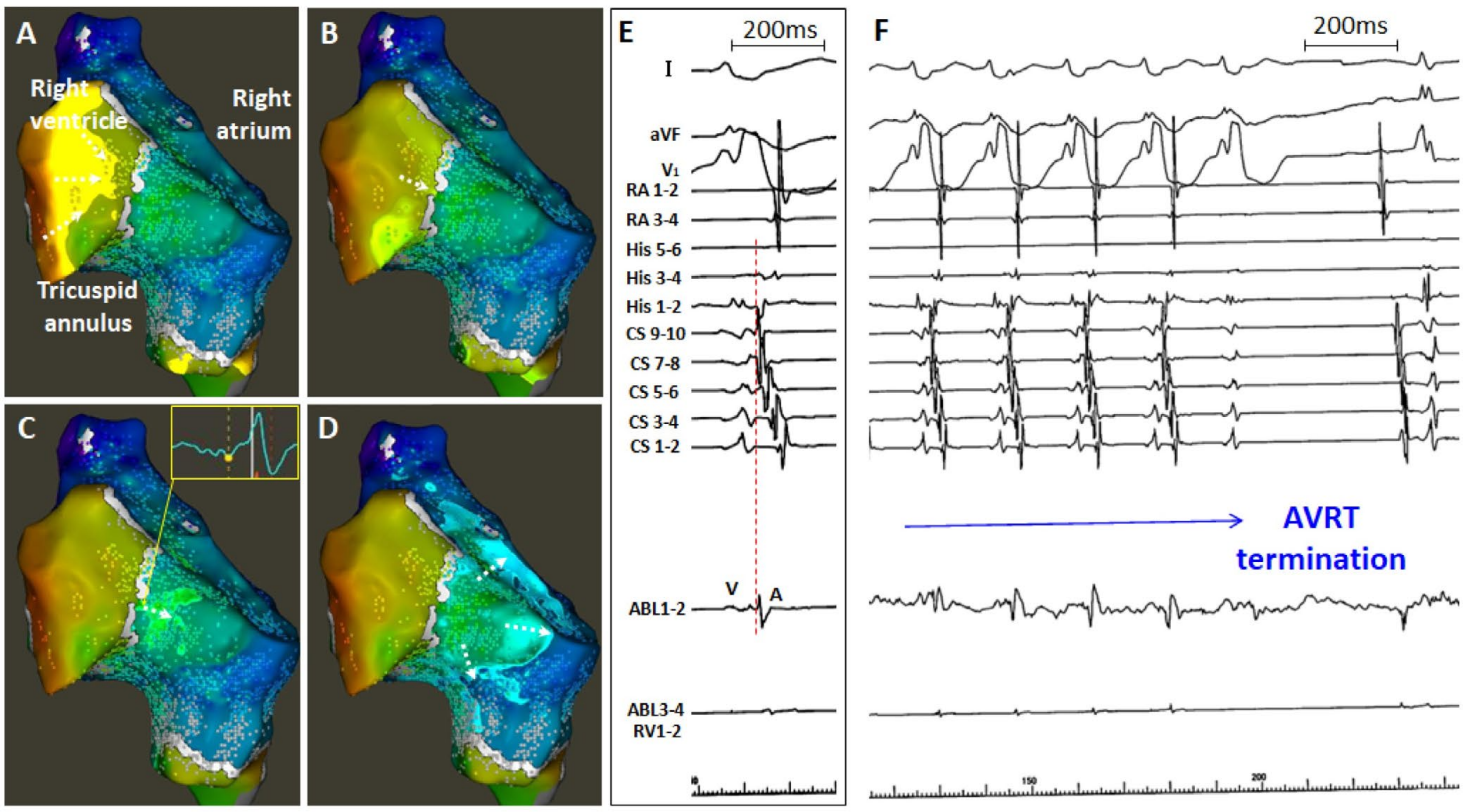
Representative case of open‐window mapping using an 8‐spline high‐density catheter (Octaray) in a patient with a concealed accessory pathway located at the septal tricuspid annulus. (A–D) Open‐window mapping performed during atrioventricular reentrant tachycardia identified an inferoseptal accessory pathway (AP) along the tricuspid annulus. The AP conduction gap was visualized by adjusting the lower extended early‐meets‐late (EEML) threshold to 22% to align with the propagation map (Supplementary Video 3). (E) Local electrograms recorded from the distal bipoles of the ablation catheter positioned within the EEML gap demonstrated the earliest atrial activation during tachycardia. (F) The first radiofrequency (RF) application at this site resulted in immediate elimination of AP conduction within 2 s.

Mapping was considered complete when the following criteria were satisfied: (1) a continuous EEML gap corresponding to the AP was clearly visualized along the atrioventricular annulus, and (2) the location of the EEML gap was spatially consistent with the propagation map and remained stable across consecutive beats. Mapping acquisition was not continued to achieve a predefined point density. Instead, mapping was terminated once stable visualization of the conduction interface was achieved according to the criteria above. Because mapping completeness was defined based on qualitative visualization rather than quantitative point density, differences in mapping density between catheter types reflect intrinsic differences in catheter sampling characteristics rather than differences in mapping endpoints. Representative examples demonstrating EEML gap visualization and AP localization using the 8‐spline catheter are shown in Figures 1, 2, 3.

### 2.4 Statistical Analysis

Continuous variables are presented as median and interquartile range (IQR) and compared using the Mann–Whitney *U*‐test. Categorical variables are presented as counts and percentages and compared using Fisher's exact test. All *p*‐values were calculated using two‐sided tests. A two‐sided *p* < 0.05 was considered statistically significant. Analyses were conducted using JMP software (SAS Institute, Cary, NC, USA).

## 3 Results

### 3.1 Baseline Characteristics

A total of 43 patients were included: 24 in the 8‐spline group and 19 in the 5‐spline group. Baseline clinical characteristics were comparable between groups (Table 1), including age (55 [47–63] vs. 56 [45–67] years; *p* = 0.88), sex (71% vs. 73% male; *p* = 1.000), and body mass index (22.5 [19.9–25.2] vs. 21.6 [18.9–24.3] kg/m^2^; *p* = 0.54). The proportion of manifest APs was significantly lower in the 8‐spline group (21% vs. 68%; *p* = 0.004). AP location distribution was similar between groups (tricuspid annulus: 12.5% vs. 21.1%; *p* = 0.680). Among the tricuspid annular APs, Septal Type C APs were identified in 2 patients in the 5‐spline group and none in the 8‐spline group. Left ventricular ejection fraction also did not differ significantly (65.5% [61.0–75.0] vs. 61.0% [59.5–65.5]; *p* = 0.10).

**TABLE 1:** Baseline characteristics.

	Total (*n* = 43)	8‐spline group (*n* = 24)	5‐spline group (*n* = 19)	*p*
Age, years (IQR)	56 (45–64)	55 (47–63)	56 (45–67)	0.880
Male, *n* (%)	31 (72%)	17 (71%)	14 (73%)	1.000
Body mass index, kg/m^2^ (IQR)	21.9 (19.3–24.7)	22.5 (19.9–25.2)	21.6 (18.9–24.3)	0.540
Manifest APs, *n* (%)	18 (41%)	5 (21%)	13 (68%)	0.004
APs in the tricuspid annulus, *n* (%)	7 (16.3%)	3 (12.5%)	4 (21.1%)	0.680
Ejection fraction, % (IQR)	64.0 (60.0–70.3)	65.5 (61.0–75.0)	61.0 (59.5–65.5)	0.100

Abbreviations: AP, accessory pathway; IQR, interquartile range.

### 3.2 Mapping and Ablation Outcomes

**FIGURE 4:**
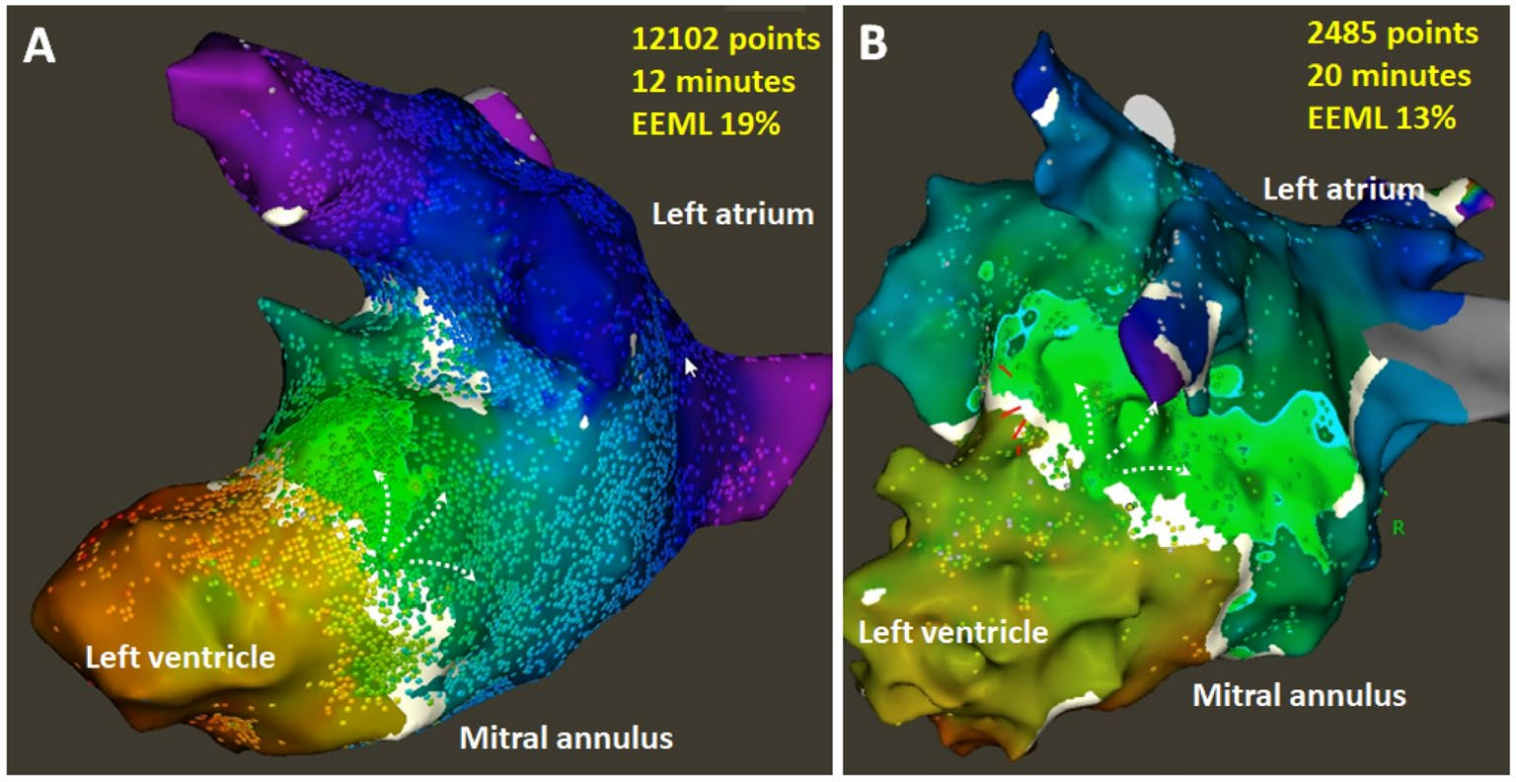
Representative comparison of open‐window mapping between 8‐spline and 5‐spline catheter designs. Representative examples of mapping density obtained using the 8‐spline catheter (Octaray) (A) and the 5‐spline catheter (Pentaray) (B) under comparable mapping durations. The 8‐spline catheter demonstrates higher point density and more detailed visualization of the extended early‐meets‐late (EEML) gap, with a narrower apparent accessory pathway (AP) width compared with the 5‐spline catheter.

The procedural characteristics are summarized in Table 2. A representative comparison of OWM between the 8‐spline and 5‐spline catheter designs is shown in Figure 4. Mapping density was markedly higher with the 8‐spline design (7974 [5618–11 017] vs. 2485 [734–12 577] points; *p* < 0.001) despite substantially shorter mapping time (11.0 [8.5–14.8] vs. 28.0 [15.0–40.0] minutes; *p* < 0.001). AP gap width derived from EEML mapping was significantly narrower in the 8‐spline group (6.1 [5.3–7.5] vs. 8.3 [2.9–15.6] mm; *p* = 0.023). Broad APs (defined as an EEML‐derived gap width ≥ 10 mm) were significantly more frequent in the 5‐spline group than in the 8‐spline group (9/19 [47.4%] vs. 1/24 [4.2%], *p* = 0.002). The lower EEML threshold required to visualize the conduction gap was also significantly smaller with the 8‐spline catheter (18.5 [16.0–20.8] vs. 23.0 [20.0–25.0]; *p* = 0.003).

**TABLE 2:** Comparison of procedural characteristics.

	8‐spline group (*n* = 24)	5‐spline group (*n* = 19)	*p*
Mapping points (IQR), points	7974 (5618–11 017)	2485 (734–12 577)	< 0.001
Mapping time, min (IQR)	11.0 (8.5–14.8)	28.0 (15.0–40.0)	< 0.001
Median AP width, mm (IQR)	6.1 (5.3–7.5)	8.3 (2.9–15.6)	0.023
Broad APs (EEML gap ≥ 10 mm), *n* (%)	1 (4.2%)	9 (47.4%)	0.002
First‐pass AP elimination, *n* (%)	24 (100%)	13 (68%)	0.004
Number of RF applications (IQR)	1.0 (1.0–1.0)	1.0 (1.0–2.0)	0.048
Lower threshold of EEML (IQR)	18.5 (16.0–20.8)	23.0 (20.0–25.0)	0.003
Fluoroscopy time, min (IQR)	7.2 (4.6–11.2)	8.9 (6.4–10.9)	0.920

Abbreviations: AP, accessory pathway; EEML, extended‐early‐meets‐late; IQR, interquartile range; RF, radiofrequency.

First‐pass AP elimination was significantly higher in the 8‐spline group (24/24, 100%) compared with the 5‐spline group (13/19, 68%; *p* = 0.004). The number of RF applications required for complete AP elimination was lower in the 8‐spline group (1.0 [1.0–1.0] vs. 1.0 [1.0–2.0]; *p* = 0.048). Fluoroscopy time was similar between groups (7.2 [4.6–11.2] vs. 8.9 [6.4–10.9] minutes; *p* = 0.92). Total procedure time was not compared due to concomitant procedures in a subset of patients. No procedural complications occurred in either group. During a median follow‐up of 12 months, no AP recurrence occurred in the 8‐spline group, whereas one recurrence occurred in the 5‐spline group (*p* = 0.442).

## 4 Discussion

This study demonstrates that OWM performed with an 8‐spline high‐density mapping catheter was associated with improved mapping performance and more efficient ablation compared with a conventional 5‐spline catheter. The principal findings are as follows: (1) the 8‐spline catheter achieved higher mapping density within a shorter mapping time, (2) AP gap delineation was more consistent and spatially confined, and (3) first‐pass ablation success was higher. These findings suggest that catheter design characteristics—including spline configuration, electrode size, and inter‐electrode spacing—may influence mapping fidelity and procedural performance during OWM‐guided AP ablation.

### 4.1 Mapping Efficiency and Catheter Design

The 8‐spline catheter acquired substantially more mapping points in a shorter time compared with the 5‐spline catheter. This improvement likely reflects design features such as increased spline number, smaller electrodes, and closer inter‐electrode spacing, which may enhance both spatial sampling density and near‐field signal resolution. High‐density point acquisition is particularly important in OWM, where accurate delineation of the EEML gap depends on resolving subtle local electrograms along the atrioventricular annulus. The higher mapping density observed with the 8‐spline catheter may facilitate clearer visualization of conduction boundaries and more reliable identification of the AP insertion site. Accordingly, the EEML‐derived AP gap width was narrower in the 8‐spline group, which may reflect improved spatial resolution rather than true anatomical differences. The finer electrode configuration may allow the EEML algorithm to operate at lower threshold settings while maintaining stable gap visualization. This increased sensitivity may more accurately reflect the true extent of the conduction interface.

The EEML threshold was adjusted on a per‐patient basis to align the EEML gap with the propagation map and optimize visualization of the conduction interface. Because electrogram characteristics, conduction velocity, rhythm stability, and far‐field contamination vary across patients and mapping conditions, a fixed threshold may not consistently represent the conduction interface. Thus, threshold selection in this study was intended to enhance interpretability rather than provide a standardized quantitative parameter. We acknowledge that non‐standardized threshold selection may influence the apparent size of the EEML gap. Therefore, the narrower AP gap observed with the 8‐spline catheter should be interpreted as reflecting improved mapping fidelity rather than true anatomical narrowing. Future software developments incorporating automated threshold optimization may improve reproducibility and allow more standardized comparisons across patients and centers. Previous studies have reported that broader APs are associated with lower first‐pass success [8]. Our findings suggest that high‐density mapping may reduce overestimation of AP width due to limited spatial resolution, potentially improving differentiation between truly broad pathways and those appearing broad because of sparse sampling. In addition, broad APs (defined as an EEML‐derived gap width ≥ 10 mm) were more frequently observed in the 5‐spline group (9 patients) than in the 8‐spline group (1 patient). This imbalance may have contributed to the lower first‐pass ablation success observed in the 5‐spline group, in addition to potential differences related to catheter design. Because EEML‐derived AP width is influenced by spatial resolution and threshold‐dependent gap delineation, the observed difference in broad AP prevalence may not necessarily reflect true anatomical differences.

### 4.2 Impact on Ablation Outcomes

The most clinically notable observation was the higher first‐pass RF application success in the 8‐spline group (100% vs. 68%). This difference was observed alongside higher mapping density and shorter acquisition time, suggesting that enhanced spatial sampling may facilitate more precise identification of the EEML‐defined conduction interface and improve targeting accuracy. Given the sequential (time‐based) adoption of the 8‐spline catheter, these findings should be interpreted as an association rather than evidence of causality. Although mapping workflow and annotation settings remained consistent during the study period, and attending oversight was maintained, residual temporal and operator‐related influences cannot be excluded. Nevertheless, catheter architecture may have contributed to improved procedural efficiency. First‐pass success is associated with reduced lesion delivery and potentially lower cumulative myocardial injury.

Regarding lesion durability, no recurrences occurred in the 8‐spline group compared with one recurrence in the 5‐spline group; however, this difference was not statistically significant. Because AP recurrence is influenced by multiple factors, including lesion quality, contact stability, and pathway anatomy, the present data do not allow definitive conclusions regarding long‐term durability. Nonetheless, improved delineation of the EEML‐defined conduction interface may facilitate more complete coverage of the functional pathway insertion, particularly in anatomically complex substrates. Previous reports have highlighted the utility of OWM in challenging settings, including redo ablations and congenital anomalies [16, 17, 18]. Whether enhanced sampling geometry translates into improved long‐term durability warrants further investigation in larger prospective studies.

### 4.3 Clinical Implications

These findings underscore the importance of sampling geometry in automated electro‐anatomical mapping. EEML‐based OWM relies on accurate visualization of the conduction interface between early‐ and late‐activated tissue. Catheter architecture—through spline number, electrode size, and inter‐electrode spacing—may influence spatial sampling density and signal fidelity. Rather than attributing procedural outcomes solely to device type, these results support a mechanistic framework in which enhanced spatial resolution may facilitate more precise lesion targeting. As mapping algorithms evolve toward increasingly automated substrate delineation, the structural characteristics of mapping catheters may play an important role in optimizing electro‐anatomical fidelity. Future prospective studies incorporating standardized threshold optimization and objective mapping fidelity metrics are warranted to further clarify the relationship between catheter design and procedural outcomes (Figures 1, 2, 3, 4).

## 5 Limitations

This study has several limitations. First, it was a retrospective, single‐center analysis with a relatively small sample size, which may limit generalizability. Second, mapping completeness was defined based on qualitative visualization of the conduction interface rather than a predefined target mapping density. Although this approach reflects real‐world clinical practice, it may have resulted in differences in point acquisition between catheter types. In particular, additional point acquisition in the 5‐spline group might have increased mapping density and potentially improved spatial resolution. However, in this study, mapping was terminated once stable visualization of the EEML‐defined conduction interface was achieved, and therefore differences in mapping density likely reflect intrinsic catheter sampling characteristics rather than differences in mapping endpoints. The TrueRef setting was available only for the 8‐spline catheter and not for the 5‐spline catheter. Therefore, differences in reference configuration may have influenced mapping accuracy and annotation stability between groups. Third, catheter allocation followed a sequential temporal adoption pattern rather than randomized assignment; therefore, potential time‐dependent confounding, including operator learning‐curve effects, cannot be fully excluded. Although multiple operators performed procedures, consistent attending oversight, unchanged mapping workflow, and stable annotation settings throughout the study period may have mitigated, but not eliminated, such bias. Manual re‐annotation was performed when necessary; however, because the extent of manual adjustment was not systematically quantified, a potential influence of operator‐dependent annotation cannot be fully excluded. Nevertheless, such adjustments were limited and unlikely to have altered the overall spatial pattern of the EEML‐derived conduction interface. Fourth, the lower EEML threshold was individualized to optimize visualization of the conduction interface by aligning the EEML gap with the propagation map. Although a standardized adjustment approach was used, this process involves operator‐dependent judgment and may influence the apparent size of the EEML‐derived gap. Therefore, the EEML‐derived AP gap should be interpreted as a functional representation of the conduction interface rather than a direct anatomical measurement. Fifth, mapping under different activation conditions (antegrade versus retrograde conduction) may introduce variability in electrogram characteristics and EEML gap appearance. Sixth, a subset of patients underwent concomitant atrial fibrillation ablation during the same procedure, which precluded a consistent comparison of total procedure time across groups. Therefore, mapping time was used as a surrogate marker of procedural efficiency in this study. Finally, this study did not compare the 8‐spline catheter with other contemporary high‐density mapping technologies. Prospective multicenter studies with standardized mapping protocols are warranted to validate these findings and further clarify the relationship between catheter sampling geometry and procedural outcomes.

## 6 Conclusion

The 8‐spline high‐density catheter was associated with improved mapping performance and more consistent visualization of accessory pathway location and extent. These findings suggest that catheter architecture may play an important role in optimizing mapping‐guided ablation strategies.

## Author Contributions

K.I.: conceptualization, data curation, formal analysis, investigation, writing – original draft. A.Y.: conceptualization, supervision, writing – review and editing. A.I.: data curation, investigation. M.A.: data curation, investigation. Y.I.: data curation, investigation. K.Y.: data curation, investigation.

## Funding

The authors have nothing to report.

## Ethics Statement

This study was conducted in accordance with the principles of the Declaration of Helsinki regarding investigations in humans and was approved by the Institutional Ethics Committee of Tokai University Hospital (approval no. 25R135).

## Conflicts of Interest

Drs. Yagishita and Iimura have received honoraria from Medtronic and Johnson & Johnson outside the submitted work. All other authors declare no conflicts of interest.

## Supporting information


**Supplementary Video 1.** Open‐window mapping of a concealed left posterolateral accessory pathway using an 8‐spline high‐density mapping catheter (Octaray). Mapping was performed during left ventricular pacing. The lower extended early‐meets‐late (EEML) threshold was adjusted to 19% to align the EEML‐derived gap with the propagation map, allowing visualization of the accessory pathway conduction interface along the mitral annulus.

**Supplementary Video 2.** Open‐window mapping of a concealed lateral tricuspid annular accessory pathway using an 8‐spline high‐density mapping catheter (Octaray). Mapping was performed during right ventricular pacing. The lower EEML threshold was adjusted to 22% to align the EEML‐derived gap with the propagation map, demonstrating oblique ventriculoatrial conduction across the tricuspid annulus.

**Supplementary Video 3.** Open‐window mapping of an inferoseptal tricuspid annular accessory pathway using an 8‐spline high‐density mapping catheter (Octaray). Mapping was performed during atrioventricular reentrant tachycardia. The lower EEML threshold was adjusted to 22% to align the EEML‐derived gap with the propagation map, allowing visualization of the accessory pathway conduction interface along the tricuspid annulus.

## Data Availability

The data supporting the findings of this study are available from the corresponding author upon reasonable request.
